# Predictive Modeling of Lignocellulosic Content in Crop Straws Using NIR Spectroscopy

**DOI:** 10.3390/plants14101430

**Published:** 2025-05-10

**Authors:** Yifan Zhao, Yingying Zhu, Yumeng Ren, Yu Lu, Chunling Yu, Geng Chen, Yu Hong, Qian Liu

**Affiliations:** 1 Faculty of Maritime and Transportation, Ningbo University, Ningbo 315211, China; 2211120087@nbu.edu.cn (Y.Z.); 2211120035@nbu.edu.cn (Y.R.); 15978581833@163.com (Y.L.); yuchunling@nbu.edu.cn (C.Y.); chengeng@nbu.edu.cn (G.C.); 2 Ningbo Key Laboratory of Green Shipping Technology, Ningbo 315211, China; 3 Ningbo Energy Group Co., Ltd., Ningbo 315042, China; 4 Ningbo Development & Investment Group, Ningbo 315042, China; 5 Faculty of Energy and Environment, Southeast University, Nanjing 211100, China; liuqian@seu.edu.cn

**Keywords:** near-infrared spectroscopy, lignocellulose, partial least squares, support vector machine

## Abstract

This study employs near-infrared spectroscopy (NIRS) combined with chemometrics to explore the feasibility and methodology for the rapid analysis of lignocellulosic content in straw. As the demand for biofuels and bioproducts increases, the efficient utilization of agricultural waste, such as straw, has become particularly important. Rapid analysis of lignocellulosic content helps improve the resource utilization efficiency of agricultural waste, providing significant support for biofuel production, agricultural waste valorization, and environmental protection. A total of 148 straw samples were used in this study, collected from Zhejiang, Jiangsu, and Heilongjiang provinces in China, covering rice straw (*Oryza sativa* L.), corn straw (*Zea mays* L.), wheat straw (*Triticum aestivum* L.), soybean straw (*Glycine max* L.), sorghum straw (*Sorghum bicolor* L.), rapeseed straw (*Brassica napus* L.), and peanut straw (*Arachis hypogaea* L.). After collection, the samples were first air-dried until surface moisture evaporated and then ground and sifted before being numbered and sealed for storage. To ensure the accuracy of the experimental results, all samples were subjected to a 6 h drying treatment at 60 °C before the experiment to ensure uniform moisture content. Partial least squares (PLS) and support vector machine (SVM) regression methods were employed for modeling analysis. The results showed that NIRS in combination with PLS modeling outperformed SVM in the calibration and prediction of lignocellulosic content. Specifically, the cellulose PLS model achieved a prediction set coefficient of determination (R^2^_P_) of 0.8983, root mean square error of prediction (RMSEP) of 0.6299, and residual predictive deviation (RPD) of 3.49. The hemicellulose PLS model had an R^2^_P_ of 0.7639, RMSEP of 1.5800, and RPD of 2.11, while the lignin PLS model achieved an R^2^_P_ of 0.7635, RMSEP of 0.6193, and RPD of 2.17. The results suggest that NIRS methods have broad prospects in the analysis of agricultural waste, particularly in applications related to biofuel production and the valorization of agricultural by-products.

## 1. Introduction

With the increasing demand for biofuels and bioproducts, efficient utilization of agricultural waste such as straw has become particularly important. However, the diversity of straw types and sources along with its slow degradation present significant challenges for its resource utilization. This study aims to analyze the lignocellulose content in straw using near-infrared spectroscopy (NIRS) technology in order to improve the resource utilization efficiency of straw and provide support for biofuel production and the valorization of agricultural waste.

The materials used in this study are biomass straws, primarily derived from crops such as rice, cron, wheat, and rapeseed. Biomass straw, as a renewable resource, holds significant potential for various applications due to its abundance and widespread availability. These straws, often considered agricultural waste, are produced in large quantities in agricultural regions, particularly in China. Through thermochemical conversion [1,2] or fermentation [3], straw can be transformed into biofuels, helping to reduce reliance on fossil fuels and minimize environmental pollution. Given the increasing demand for biofuels and the need to optimize agricultural waste utilization, exploring the lignocellulosic composition of straw is essential. This study aims to enhance the resource efficiency of straw, providing valuable insights for biofuel production and contributing to the sustainable valorization of agricultural waste. Accurately determining the lignocellulose content in biomass straw is critical, as it directly impacts its potential applications in the biomass energy and materials sector. Hemicellulose, interwoven with cellulose to form the fundamental structure of the cell wall [4,5], provides a diverse carbon source [6,7] during decomposition, representing a critical step in biomass energy conversion. Lignin imparts unique mechanical strength [8] and corrosion resistance [9] to straw, with its high chemical resistance and biodegradability holding promise for applications in biomass materials. Accurate research into lignocellulose content is essential for optimizing biomass energy production processes, expanding the application range of biomass materials, addressing the technical challenges in biomass straw utilization, and facilitating broader and more efficient deployment in the energy and materials sectors.

Currently, many researchers employ the following methods to investigate the content of cellulose, hemicellulose, and lignin. Barta-Rajnai et al. [10] investigated the pyrolysis of *locust wood* (*Robinia pseudoacacia* L.), wheat, and rapeseed straw at various temperatures. They determined the content of cellulose, hemicellulose, and Klason lignin through compositional analysis and acid hydrolysis. Benouadah et al. [11] utilized acid methanol hydrolysis and gas chromatography (GC) to determine the hemicellulose content. Zhang et al. [12] developed a novel green lignin quantification method, utilizing a LiCl/DMSO solution to dissolve lignocellulosic biomass and determining lignin content through a UV spectrophotometric technique known as the LiCl/DMSO CDS-UV method. Overall, it is crucial to develop a high-efficiency, eco-friendly technology for assessing lignocellulose content. Although traditional methods are effective, they often require high temperatures, chemicals, and complex procedures. In contrast, this study uses near-infrared spectroscopy (NIRS), an efficient, eco-friendly, and non-destructive technique [13,14]. NIRS enables rapid and accurate analysis of lignocellulose in straw samples without the need for complex reagents or high temperatures, offering a simpler and more adaptable approach for large-scale screening and real-time monitoring [15].

The technique for detecting lignocellulosic content using NIRS has reached a mature stage. For example, Yin et al. [16] used tobacco (*Nicotiana tabacum* L.) as a research subject, employing NIRS to characterize the biomass pyrolysis process. Xue et al. [17] conducted online near-infrared detection of lignocellulose content in corn straw. Zhang et al. [18] used NIRS to measure cellulose and hemicellulose content in big bluestem (*Andropogon gerardii Vitman*), advancing research in plant breeding and genetics. In addition to biomass energy [19,20,21], NIRS finds applications in a wide range of fields, including food [22,23], agriculture [24], plants [25], medicine [26], and environmental monitoring [27,28,29]. NIRS has proven to be an effective tool in these fields, with numerous studies and applications demonstrating its potential.

However, NIRS is not without its limitations. One major drawback is its sensitivity to external environmental conditions, particularly soil moisture, which can significantly reduce measurement accuracy [30]. Additionally, when NIRS is used for monitoring tissue oxygenation, various physiological and non-physiological factors may interfere, leading to errors in interpretation of the results [31]. Moreover, NIRS imaging systems face challenges related to image quality, localization accuracy, and system stability, which may affect the overall performance of the technology [32]. Despite these limitations, the robustness and speed of NIRS combined with advanced modeling techniques make it a highly valuable tool for lignocellulosic content analysis.

While NIRS has matured as a technique for lignocellulose analysis, the novelty of this study lies in the combination of NIRS with two advanced modeling approaches, partial least squares (PLS) and support vector machines (SVM), to fully explore the lignocellulose content in straw. Additionally, four wavelength screening methods were employed after pretreatment to optimize the analysis, ensuring the robustness and accuracy of the results. Evaluation criteria, including root mean square error (RMSE), coefficient of determination (R^2^), and residual predictive deviation (RPD), were used to select the best methods, offering a more efficient, adaptable, and reliable approach compared to traditional techniques.

## 2. Materials and Methods

### 2.1. Sample Preparation

The crop straws utilized in this study were predominantly obtained from the provinces of Zhejiang, Jiangsu, and Heilongjiang in China. A total of 148 samples were prepared, encompassing various parameters such as geographical origin, climate conditions, and crop varieties. The specific crop varieties used in this study included rice, corn, wheat, soybean, sorghum, rapeseed, and peanut. The samples were uniformly dried to remove surface moisture and then ground using a 40-mesh sieve. To prevent moisture absorption during processing, which could compromise the accuracy of NIRS analysis, minimizing air exposure is critical. The samples were then dried in a controlled environment in a blower drying oven set at 60 ± 5 °C for 6 h to ensure consistent moisture content before experimentation. After drying, the samples were allowed to cool naturally and stored in sealed bags at room temperature for 3 weeks before spectral analysis. Moisture content verification post-drying was performed by calculating the weight difference before and after the drying process. The samples were protected from moisture during storage to maintain consistency and ensure the reliability of the analysis results.

### 2.2. Spectral Data Processing and Analysis

#### 2.2.1. Spectra Acquisition and Abnormal Sample Elimination

In this experiment, we employed the NEXUS670 near-infrared spectrometer (manufactured by Thermo Fisher Scientific, Waltham, MA, USA) to capture the spectra of the straw samples. Given that the samples were in solid powder form, we selected the diffuse reflection mode for spectral scanning. The wavelength range was configured from 10,000 to 4000 cm^−1^, corresponding to 1000 to 2500 nm, with a resolution of 8 cm^−1^. To ensure precision, we conducted 32 measurements for each sample, and the resultant average spectrum was recorded for further analysis.

The presence of anomalous samples In NIRS often stems from errors or substantial deviations within spectral data. Such deviations may result from instrument inaccuracies, improper sample handling, or variations in experimental conditions. To mitigate their impact on regression analysis, this study employs principal component analysis combined with the Mahalanobis distance method (PCA–MD) for screening purposes. This approach facilitates the identification and exclusion of potential outliers, thereby enhancing the accuracy and reliability of the analysis. During screening, a threshold for the MD of samples is established. Samples exceeding this threshold are deemed abnormal spectral and consequently discarded [33]. In the selection of the threshold value, we utilized the “e” value of 1.5 because of the presence of abnormal samples leading to an elevated standard deviation. A lower threshold value was essential to effectively eliminate these samples in our investigation [34]. After undergoing screening through the PCA–MD method, four samples were identified as outliers and subsequently removed.

#### 2.2.2. Sample Division

Selecting an appropriate calibration set is crucial for ensuring the predictive accuracy of the model. An optimal calibration set should encompass the full spectrum of physical and chemical parameters inherent in the sample sets. The concentration levels must cover all parameters observed in the validation set samples, and the physical and chemical parameters within the calibration set must be evenly distributed. Moreover, the sample size must be sufficient to establish a robust functional relationship between spectral data and the physical and chemical parameters. In this study, the sample set partitioning based on joint x–y distances (SPXY) method was utilized for partitioning the sample sets. The SPXY method improves the Kennard–Stone (K–S) algorithm by incorporating both spectral (X) and chemical (Y) data in the sample selection process, optimizing the calibration set and enhancing model performance.

#### 2.2.3. Spectral Preprocessing

NIRS data contain valuable information on sample characteristics as well as baseline drift and spectral noise caused by various unrelated factors, such as light scattering and instrument response. Thus, preprocessing the raw spectrum is essential. Effective preprocessing techniques mitigate interference, enhance resolution, and simplify prediction models [35]. In quantitative calibration models for biological samples via NIRS, preprocessing plays a pivotal role. Selecting appropriate methods minimizes environmental noise impact, thereby improving predictive capability and sample analysis quality. Savitzky–Golay smoothing (SG) relies on comparing the fitting residuals, which represent the differences between the input signal and the smoothed signal with the inherent noise of the instrument [36]. The first-order derivative (FD) can remove linear-independent drift, while the second-order derivative (SD) can eliminate linear-dependent drift [37]. Standard normal variate (SNV) and multiplicative scatter correction (MSC) are effective methods for mitigating signal variations; they are commonly used in NIRS to correct slope variability and scatter-related effects [38]. This study utilized preprocessing methods, including SG, FD, SD, SNV, and MSC.

#### 2.2.4. Characteristic Wavelength Screening

NIRS data acquired by near-infrared spectrometers often have numerous wavelength points and cover a wide range of bands. While this data contain valuable physical and chemical information about the sample, the data also include noise and redundant information that can hinder the establishment of accurate prediction models for the sample’s properties. Even with preprocessing methods applied for denoising, ensuring high accuracy and stability of the prediction model remains a challenge. Spectral feature wavelength selection methods are crucial for eliminating irrelevant and overlapping variables and identifying the variables that contain valuable characteristic information. This process helps reduce the computational complexity of the modeling process and improves the accuracy and robustness of predictive models. Several methods can be used to select characteristic wavelengths in NIRS, including the successive projections algorithm (SPA), genetic algorithm (GA), competitive adaptive reweighted sampling (CARS), and interval variable iterative space shrinkage approach (IVISSA).

### 2.3. Modeling Method

We utilized partial least squares (PLS) regression and support vector regression (SVR) for constructing our prediction models. PLS is a robust method for multiple linear regression, particularly adept at handling collinearity issues present in spectral curves during modeling [39]. Conversely, SVR is suitable for solving nonlinear, small-sample, high-dimensional modeling and pattern recognition problems, but its prediction accuracy is related to the penalty coefficient and radial basis function kernel parameter [40].

### 2.4. Model Evaluation

Following the construction of the model, it is imperative to evaluate its performance using various parameters. Typically, the calibration model is established using data from a dedicated calibration set, then validated against a prediction set to determine the correlation between the predicted results and chemical values acquired via standardized methodologies. This helps determine the suitability and stability of the model. *R*^2^ serves as a statistical gauge for assessing the adequacy of regression models [41]. It quantifies the proportion of variance in the dependent variable that is accounted for by the independent variables. *R*^2^ values range from 0 to 1, with higher values indicating a stronger fit between the model and the observed data. The *R*^2^ was calculated by Equation (1):

(1) $$R^{2}=1-\frac{\sum_{j=1}^{n}\left(y_{j}-\hat{y_{j}}\right)}{\sum_{j=1}^{n}{\left[y_{j}-\frac{1}{n}\sum_{j=1}^{n}y_{j}\right]}^{2}}$$

where *y_j_* denotes the actual value of a sample within the calibration set and $\hat{y_{j}}$ signifies the predicted value of the calibration set. Here, *j* represents the serial number of the sample.

RMSE is a fundamental metric in assessing the accuracy of a predictive model. It quantifies the average deviation between observed and predicted values, providing a comprehensive measure of model performance, and lower RMSE values indicate better predictive accuracy [42]. Root mean square error of calibration (*RMSEC*) evaluates model performance on calibration data, while root mean square error of prediction (*RMSEP*) assesses predictive accuracy on independent validation data. The *RMSEC* was calculated by Equation (2), and the *RMSEP* by Equation (3):

(2) $$RMSEC=\sqrt{\frac{1}{n}\sum_{j=1}^{n}(y_{j}-\hat{y_{j}}{)}^{2}}$$

(3) $$RMSEP=\sqrt{\frac{1}{n^{k}}\sum_{j=1}^{n^{k}}({y_{j}}^{k}-{\hat{y_{j}}}^{k}{)}^{2}}$$

In addition to the *R*^2^ and RMSE, RPD was used as a performance indicator of the near-infrared models. In general, an *RPD* value is interpreted as follows: *RPD* values less than 1.5 indicate an unreliable model, values between 1.5 and 2 suggest a moderately reliable model, and values greater than 2 indicate a model with high reliability that is suitable for predictive analysis. The *RPD* was calculated by Equation (4):

(4) $$RPD=\frac{STDEV}{RMSEP}$$

where *STDEV* is the standard deviation.

## 3. Results and Discussion

### 3.1. Sample Set Division

The SPXY methodology was utilized to partition the 144 samples into a calibration set and a prediction set at a 2:1 ratio, yielding 96 samples in the calibration set and 48 samples in the prediction set, as depicted in Table 1.

### 3.2. Spectral Data Preprocessing

#### 3.2.1. PLS Prediction Models Based on Full Spectrum

Table 2 presents the prediction results of PLS and SVR models based on the full spectrum after preprocessing. The table illustrates PLS prediction outcomes for lignocellulose in straw samples, comparing the raw and preprocessed spectra. For cellulose, SD preprocessing yielded excellent results, with RPD and RMSEP values of 2.89 and 0.765, respectively. The hemicellulose models benefited significantly from MSC preprocessing, yielding the lowest RMSEP of 1.7114. SNV preprocessing demonstrated the highest accuracy for the lignin models, with an RMSEP of 0.6193.

#### 3.2.2. SVR Prediction Models Based on Full Spectrum

**Figure 1:**
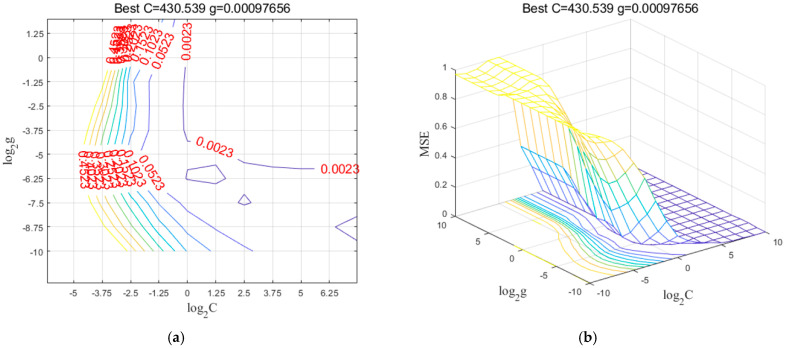
Grid search parameter c and g process: (**a**) Contour maps; (**b**) Three-dimensional view.

For SVR prediction models, the radial basis function (RBF) kernel was chosen as the kernel function for the SVR model of lignocellulose. The penalty factor c and kernel parameter g of the model were determined through an iterative process using grid search (GS) combined with cross-validation. The parameter range for c and g was set to [2^−10^, 2^10^]. Figure 1a,b display the contour plot and 3D view, respectively, of the optimal parameters for the cellulose SVR model obtained through the grid search method. The optimal penalty factor c was found to be 430.53, while the kernel parameter g was determined to be 9.76 × 10^−4^.

Table 2 summarizes SVR prediction models for lignocellulose in untreated and preprocessed biomass straw samples. Among the preprocessing methods, MSC yielded the best predictive performance for cellulose prediction, with an RMSEP value of 1.0521, and R^2^_P_ and RPD values of 0.7938 and 2.09, respectively. The SD preprocessing yielded the best predictions for hemicellulose, with the highest R^2^_P_ and RPD values at 0.6329 and 1.59, respectively, and the lowest RMSEP of 2.1159. For lignin prediction, only SNV preprocessing outperformed the original model, with R^2^_P_ and RPD values of 0.5735 and 1.67, respectively, and an RMSEP of 0.8448.

### 3.3. Prediction Models for Lignocellulose Based on Characteristic Wavelengths

After preprocessing, the characteristic wavelengths were selected using the CARS, SPA, GA, and IVISSA algorithms. The optimal penalty factor c and kernel parameter g were determined through the grid search method. Subsequently, PLS and SVR prediction models were developed based on the selected characteristic wavelengths to predict lignocellulose content.

#### 3.3.1. PLS Prediction Model for Lignocellulose Based on Characteristic Wavelengths

The predicted results are presented in Table 3. For predicting cellulose content, the PLS model built using the full-wavelength vectors achieved an R^2^_P_ of 0.8578, an RMSEP of 0.765, and an RPD of 2.89. Nonetheless, the models utilized three characteristic wavelengths, the SPA, GA, and IVISSA algorithms, which exhibited inferior predictive performance compared to the full-wavelength model. Specifically, for the SPA combined with PLS model, the R^2^_P_ and RPD decreased by 0.4006 and 1.11, respectively, while the RMSEP increased by 0.4689. On the other hand, the CARS combined with PLS model, built using 68 eigenvectors, achieved an R^2^_P_ of 0.8983, an RMSEP of 0.6299, and an RPD of 3.49, with an increase in R^2^_P_ and RPD by 0.0405 and 0.6, respectively, and a decrease in RMSEP by 0.1351 compared to the full-wavelength PLS model. After evaluating the overall efficacy of four PLS models using different feature-wavelength selection methods, the CARS algorithm demonstrated the best predictive performance for cellulose content. Consequently, the CARS algorithm would be employed in the cellulose content PLS prediction model.

For hemicellulose content prediction, we developed a full-wavelength PLS model using 1556 wavelength vectors and applied the MSC method for spectral preprocessing. The model achieved an R^2^_P_ of 0.7465, an RMSEP of 1.7114, and an RPD of 1.96, with six principal factors. For the hemicellulose PLS models built with the SPA, IVISSA, and GA algorithms, the prediction results were lower compared to the full-wavelength model. On the other hand, the CARS combined with PLS model, built from 35 eigenvectors, outperformed the full-wavelength model with an R^2^_P_ of 0.7639, an RMSEP of 1.58, and an RPD of 2.11, with an increase of 0.0174 in R^2^_P_ and 0.15 in RPD and a decrease of 0.1314 in RMSEP compared to the full-wavelength model. Hence, for the hemicellulose PLS model, the CARS algorithm demonstrated the best predictive performance for hemicellulose content. Consequently, the CARS algorithm would be employed in the hemicellulose PLS prediction model.

For lignin content prediction, a full-wavelength PLS model was developed using the SNV pretreatment method. With seven main factors, the model achieved an R^2^_P_ of 0.7635, an RMSEP of 0.6193, and an RPD of 2.17. Upon comparing the predictive outcomes of the four characteristic wavelength models with the full-wavelength model, a consistent trend emerged: the characteristic wavelength-based models consistently exhibited inferior performance compared to the full-wavelength model. Consequently, for the lignin PLS prediction model, the decision was made to develop a correction model utilizing the full-wavelength vector.

#### 3.3.2. SVR Prediction Model for Cellulose, Hemicellulose, and Lignin Based on Characteristic Wavelength

The predicted results are presented in Table 4. For cellulose prediction, the CARS, SPA, IVISSA, and GA algorithms were utilized to select characteristic wavelength vectors and construct corresponding SVR cellulose prediction models. Upon comparing the prediction results, it was observed that, with the exception of the SPA combined with SVR model, the other three characteristic wavelength SVR models outperformed the full-wavelength model. Among them, the CARS combined with SVR model exhibited the most accurate predictions, with an increase of 0.0993 and 0.92 in R^2^_P_ and RPD, respectively, and a decrease of 0.2486 in RMSEP compared to the full-wavelength SVR model. The SPA algorithm performed the worst, with a decrease of 0.0985 and 0.33 in R^2^_P_ and RPD, respectively, and an increase of 0.2156 in RMSEP compared to the full-wavelength SVR model. Thus, for the cellulose SVR model, the CARS algorithm demonstrated the best predictive performance for hemicellulose content.

For hemicellulose prediction, the GA and IVISSA algorithms all performed worse than the full-wavelength model. Specifically, the SPA combined with SVR model showed an increase of 0.0117 and 0.05 in R^2^_P_ and RPD, respectively, and a decrease of 0.1566 in RMSEP compared to the full-wavelength SVR model. Therefore, the hemicellulose SVR model was developed using the SPA modeling approach.

For lignin prediction, the SVR models built from the characteristic wavelengths screened by GA and IVISSA performed worse than the full-wavelength models. In contrast, the lignin SVR models constructed with feature wavelengths selected by CARS and SPA outperformed the full-wavelength model in terms of predictive performance. In particular, the predictive performance of the CARS method exceeded that of SPA. Compared to the full-wavelength SVR model, the CARS combined with SVR model achieved increases of 0.0904 in R^2^_P_ and 0.05 in RPD, while RMSEP decreased by 0.0978. Therefore, the CARS method was used to construct the lignin SVR model.

#### 3.3.3. Comparison of PLS and SVR Model Effects

The CARS, SPA, IVISSA, and GA wavelength extraction algorithms were combined with PLS models to create predictive models for the lignocellulosic content in crop straw. The cellulose model had R^2^_P_ values ranging from 0.4572 to 0.8983, RMSEP values ranging from 0.6299 to 1.2339, and RPD values ranging from 1.78 to 3.49. The hemicellulose model had R^2^_P_ values ranging from 0.5554 to 0.7639, RMSEP values ranging from 1.5800 to 2.26, and RPD values ranging from 1.43 to 2.11. The lignin model had R^2^_P_ values ranging from 0.2414 to 0.7635, RMSEP values ranging from 0.6193 to 0.8626, and RPD values ranging from 1.49 to 2.17.

The CARS, SPA, IVISSA, and GA wavelength selection algorithms were integrated with SVR models to develop predictive models for measuring cellulose, hemicellulose, and lignin content in crop straws. The cellulose model had R^2^_P_ values ranging from 0.6953 to 0.8931, RMSEP values ranging from 0.8035 to 1.2677, and RPD values ranging from 1.76 to 3.01. The hemicellulose model had R^2^_P_ values ranging from 0.4930 to 0.6552, RMSEP values ranging from 1.0731 to 2.2500, and RPD values ranging from 1.20 to 1.64. The lignin model had R^2^_P_ values ranging from 0.4278 to 0.6639, RMSEP values ranging from 0.7470 to 1.5060, and RPD values ranging from 1.12 to 1.72.

**Figure 2:**
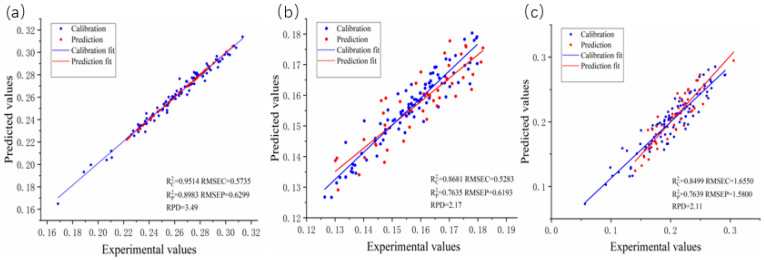
The scatter plots of chemical values and predicted values of the optimal prediction model for straw biomass lignocellulose: (**a**) Cellulose; (**b**) Hemicellulose; (**c**) Lignin.

Figure 2 displays the scatter plots of the chemical values and predicted values from the optimal prediction model for crop straw lignocellulose. Figure 2a shows a scatter plot of the PLS prediction model for cellulose content. Figure 2b illustrates a scatter plot for the PLS prediction model for hemicellulose. Finally, Figure 2c presents a scatter plot for the PLS prediction model for lignin.

Among all the prediction models, the cellulose PLS model demonstrated exceptional accuracy, boasting an impressive R^2^_P_ value of 0.8983, an RMSEP of 0.6299, and an RPD value of 3.49. The combination of the SD and CARS methods with PLS models proved to be the most effective approach for predicting cellulose. The cellulose model demonstrated excellent performance, with an R^2^_P_ of 0.8983, RMSEP of 0.6299, and RPD of 3.49, indicating strong predictive power, low error, and high reliability. These metrics meet the ideal standards (R^2^_P_ > 0.9, RMSEP < 1%, RPD > 2).

However, the PLS and SVR models fell short when it came to accurately predicting the lignin. The SNV combined with PLS model emerged as the top choice for lignin prediction, delivering an R^2^_P_ value of 0.7635, an RMSEP value of 0.6193, and an RPD value of 2.17. The lignin model showed good performance, with an R^2^_P_ of 0.7635, RMSEP of 0.6193, and RPD of 2.17. Although its R^2^_P_ was slightly below the optimal range (0.8–0.9), it remained reliable for predictions with moderate error.

In the case of hemicellulose, the characteristic wavelength processing of the PLS and SVR models did not yield significantly improved predictive performance. The optimal prediction model for hemicellulose involved the combination of MSC and CARS with PLS, yielding an R^2^_P_ value of 0.7639, an RMSEP value of 1.58, and an RPD value of 2.11. The hemicellulose model, with an R^2^_P_ of 0.7639, RMSEP of 1.58, and RPD of 2.11, exhibited moderate accuracy, but its higher RMSEP suggests potential for further refinement. Overall, the cellulose model is the most robust, while the lignin and hemicellulose models, though usable, could benefit from additional optimization.

### 3.4. Comparison and Discussion

Compared to the iPLS-based models developed by Ai et al. [43] for *Sargassum horneri*, our cellulose and lignin models outperformed theirs, with higher R^2^_P_ and RPD values, while the hemicellulose model exhibited slightly lower accuracy. Zhou et al. [44] investigated the rapid determination of cellulose content in pulp using NIRS, and their cellulose results align closely with ours. However, Zhang et al. [18] developed NIR prediction models for cellulose and hemicellulose contents in big bluestem biomass, achieving better results for hemicellulose than our models. Additionally, Guo et al. [45] applied NIRS to evaluate nutritional components in corn stover and wheat straw. Their hemicellulose results showed limited reliability, making it suitable only for preliminary screening.

NIRS demonstrates strong potential for rapid assessment of cellulose and lignin in straw biomass; however, its utility for hemicellulose quantification remains limited due to spectral complexity and compositional heterogeneity. While some studies, such as those by Zhang et al. [18], have achieved better results for hemicellulose prediction, the overall performance remains below that for cellulose and lignin. Guo et al.’s [45] work also highlights the limited reliability of hemicellulose predictions, making it suitable primarily for preliminary screening.

To overcome these challenges, future studies should focus on improving spectral preprocessing methods and exploring advanced machine learning approaches. Combining multiple types of spectroscopy and tailoring models to specific species could also make NIR predictions more reliable. With these improvements, we could enhance accuracy and achieve high-precision predictions for hemicellulose, ultimately making NIR a powerful tool for lignocellulosic biorefining.

## 4. Conclusions

NIRS technology has significant potential for predicting the lignocellulosic content of crop straw. Preprocessing techniques, such as SD, MSC, and SNV, have notably enhanced prediction performance. After preprocessing, it was found that, for cellulose and hemicellulose, the CARS algorithm combined with PLS models yielded the best predictions, while for lignin, the full-wavelength PLS model provided the best results. Within the realm of NIRS, both PLS and SVR methodologies have showcased commendable calibration and prediction outcomes. Generally, PLS models offer higher predictive accuracy compared to SVR models. Additionally, the CARS method used for selecting feature wavelengths generally exhibited superior predictive performance compared to SPA, GA, and IVISSA, except for the lignin models. The results indicate that NIRS technology is effective in predicting lignocellulose content quickly and efficiently.

## Figures and Tables

**Table 1:** Statistics of lignocellulose of straw biomass based on the SPXY method.

	Sample Set	Sample Size	Max. (%)	Min. (%)	Average (%)	Standard Deviation
Cellulose	Calibration Set	96	31.32	25.85	26.01	1.78
	Prediction Set	48	30.82	22.23	26.13	1.94
Hemicellulose	Calibration Set	96	30.61	15.62	19.98	2.78
	Prediction Set	48	26.66	15.04	20.56	2.81
Lignin	Calibration Set	96	18.83	12.63	15.69	1.41
	Prediction Set	48	17.73	13.61	15.72	1.21

**Table 2:** The results of PLS and SVR prediction models for cellulose, hemicellulose, and lignin of straw biomass based on original wavelengths.

		PLS				SVR				
	Method	Factors	R^2^_P_	RMSEP	RPD	Penalty Factor c	Kernel Parameter g	R^2^_P_	RMSEP	RPD
Cellulose	None	8	0.6214	1.2498	1.77	13.45	9.76 × 10^−4^	0.6135	1.4582	1.51
	SNV	8	0.7230	1.0850	2.03	430	9.76 × 10^−4^	0.6820	1.3010	1.69
	MSC	6	0.7850	0.9617	2.30	5.65	2.32 × 10^−3^	0.7938	1.0521	2.09
	FD	7	0.8224	0.8136	2.70	13.45	4.10 × 10^−4^	0.7784	1.0780	2.04
	SD	6	0.8578	0.7650	2.89	13.45	4.10 × 10^−4^	0.7832	1.0658	2.06
	SNV + FD	8	0.5118	1.4090	1.54	13.45	4.10 × 10^−4^	0.6379	1.4104	1.56
Hemicellulose	None	11	0.5438	2.5134	1.33	13.45	9.76 × 10^−4^	0.5428	2.3441	1.43
	SNV	8	0.7077	1.9609	1.71	3.2	2.32 × 10^−3^	0.6266	2.1647	1.55
	MSC	6	0.7465	1.7114	1.96	13.45	9.76 × 10^−4^	0.4728	2.5117	1.34
	FD	10	0.5742	2.4354	1.38	13.45	4.10 × 10^−4^	0.5334	2.3740	1.42
	SD	10	0.6551	2.0838	1.61	13.45	4.10 × 10^−4^	0.6329	2.1159	1.59
	SNV + FD	9	0.5545	2.4770	1.35	2.37	2.32 × 10^−3^	0.5360	2.2203	1.51
Lignin	None	9	0.5235	0.7691	1.83	13.45	9.76 × 10^−4^	0.5457	0.8674	1.63
	SNV	7	0.7635	0.6193	2.17	7.61	9.76 × 10^−4^	0.5735	0.8448	1.67
	MSC	8	0.6863	0.6565	1.99	13.45	9.76 × 10^−4^	0.4483	0.9736	1.45
	FD	8	0.6776	0.6699	2.10	13.45	4.10 × 10^−4^	0.3926	1.0267	1.37
	SD	7	0.5310	0.7514	1.88	13.45	1.72 × 10^−4^	0.4324	0.9847	1.43
	SNV + FD	7	0.4055	0.7925	1.51	5.65	4.10 × 10^−4^	0.5186	0.8964	1.57

**Table 3:** The results of the PLS prediction model for cellulose, hemicellulose, and lignin of straw biomass based on characteristic wavelengths.

	Method	Number of Wavelength Variables	Factors	R^2^_C_	RMSEC	R^2^_P_	RMSEP	RPD
Cellulose	SD	1556	6	0.9734	0.4290	0.8578	0.7650	2.89
	SD + CARS	68	4	0.9514	05735	0.8983	0.6299	3.49
	SD + SPA	9	4	0.7608	1.1713	0.4572	1.2339	1.78
	SD + IVISSA	948	4	0.9357	0.6552	0.8267	0.7960	2.76
	SD + GA	123	6	0.9712	0.4455	0.8564	0.7747	2.84
Hemicellulose	MSC	1556	6	0.7804	1.9440	0.7465	1.7114	1.96
	MSC + CARS	35	7	0.8499	1.6550	0.7639	1.5800	2.11
	MSC + SPA	10	4	0.6304	2.3810	0.5738	1.9712	1.65
	MSC + IVISSA	115	6	0.8175	1.8241	0.5554	2.2600	1.43
	MSC + GA	1063	4	0.7521	2.0420	0.5803	2.2072	1.47
Lignin	SNV	1556	7	0.8681	0.5238	0.7635	0.6193	2.17
	SNV + CARS	52	5	0.9201	0.7643	0.6432	0.7657	1.68
	SNV + SPA	18	5	0.1256	1.0482	0.2414	0.8626	1.49
	SNV + IVISSA	973	4	0.4284	0.9255	0.3857	0.8385	1.54
	SNV + GA	196	5	0.9514	0.2656	0.5518	0.7233	1.77

**Table 4:** The results of the SVR prediction model for cellulose, hemicellulose, and lignin of straw biomass based on characteristic wavelengths.

	Method	Number of Wavelength Variables	Penalty Factor c	Kernel Parameter g	R^2^_C_	RMSEC	R^2^_P_	RMSEP	RPD
Cellulose	MSC	1556	5.65	2.32 × 10^−3^	0.8853	0.9139	0.7938	1.0521	2.09
	MSC + CARS	68	13.45	5.52 × 10^−3^	0.9337	0.7535	0.8931	0.8035	3.01
	MSC + SPA	9	5.65	1.31 × 10^−2^	0.8255	1.1899	0.6953	1.2677	1.76
	MSC + GA	123	13.45	5.52 × 10^−3^	0.9701	0.4598	0.8446	0.8948	2.53
	MSC + IVISSA	948	13.45	4.41 × 10^−4^	0.9294	0.7374	0.8175	0.9746	2.29
Hemicellulose	SD	1556	13.45	4.10 × 10^−4^	0.8966	1.5181	0.6329	2.1159	1.59
	SD + CARS	35	13.45	0.17	0.8910	1.5168	0.6552	1.9806	1.63
	SD + SPA	10	13.45	0.42	0.8347	1.8674	0.6446	1.9593	1.64
	SD + GA	115	1024	2.32 × 10^−3^	0.8430	0.6099	0.4930	1.0731	1.20
	SD + IVISSA	1063	13.45	2.32 × 10^−3^	0.8019	2.1266	0.5288	2.2500	1.44
Lignin	SNV	1556	7.61	9.76 × 10^−4^	0.5994	0.9762	0.5735	0.8448	1.67
	SNV + CARS	52	13.45	3.12 × 10^−2^	0.8059	0.7118	0.6639	0.7470	1.72
	SNV + SPA	18	2.37	7.43 × 10^−2^	0.4991	1.089	0.6176	0.7929	1.61
	SNV + GA	196	1024	2.32 × 10^−3^	0.8202	0.6559	0.4278	1.5060	1.12
	SNV + IVISSA	973	13.45	2.32 × 10^−3^	0.6966	0.8771	0.5667	0.8756	1.51

## Data Availability

All data are included within the article.

## References

[1] Lee D. (2022). Recent Progress in the Catalytic Thermochemical Conversion Process of Biomass for Biofuels. Chem. Eng. J..

[2] Ricciulli M.O., Arce G.L.A.F., Vieira E.C., Ávila I. (2024). Interaction among Lignocellulosic Biomass Components in Thermochemical Processes. Biomass Bioenergy.

[3] Müller J., Wiedow D., Chmit M.S., Beuerle T. (2022). Utilization of Biomasses from Landscape Conservation Growths Dominated by Common Ragwort (*Jacobaea Vulgaris* Gaertn.) for Biomethanization. Plants.

[4] Qaseem M.F., Shaheen H., Wu A.-M. (2021). Cell Wall Hemicellulose for Sustainable Industrial Utilization. Renew. Sustain. Energy Rev..

[5] Terrett O.M., Dupree P. (2019). Covalent Interactions between Lignin and Hemicelluloses in Plant Secondary Cell Walls. Curr. Opin. Biotechnol..

[6] Guo H., Wang X.-D., Lee D.-J. (2018). Proteomic Researches for Lignocellulose-Degrading Enzymes: A Mini-Review. Bioresour. Technol..

[7] Najjarzadeh N., Matsakas L., Rova U., Christakopoulos P. (2021). How Carbon Source and Degree of Oligosaccharide Polymerization Affect Production of Cellulase-Degrading Enzymes by *Fusarium Oxysporum* f. sp. Lycopersici. Front. Microbiol..

[8] Du B., Chen C., Sun Y., Yang M., Yu M., Liu B., Wang X., Zhou J. (2020). Unlocking the Response of Lignin Structure by Depolymerization Process Improved Lignin-Based Carbon Nanofibers Preparation and Mechanical Strength. Int. J. Biol. Macromol..

[9] Wang D. (2024). A Strong Enhancement of Corrosion and Wear Resistance of Polyurethane-Based Coating by Chemically Grafting of Organosolv Lignin. Mater. Today Chem..

[10] Barta-Rajnai E., Jakab E., Sebestyén Z., May Z., Barta Z., Wang L., Skreiberg Ø., Grønli M., Bozi J., Czégény Z. (2016). Comprehensive Compositional Study of Torrefied Wood and Herbaceous Materials by Chemical Analysis and Thermoanalytical Methods. Energy Fuels.

[11] Benouadah N., Aliouche D., Pranovich A., Willför S. (2019). Chemical Characterization of Pinus Halepensis Sapwood and Heartwood. Wood Mater. Sci. Eng..

[12] Zhang H., Zhao H., Yang Y., Ren H., Zhai H. (2022). A Spectroscopic Method for Quantitating Lignin in Lignocellulosic Biomass Based on the Completely Dissolved Solution of Biomass in LiCl/DMSO. Green Chem..

[13] Gao F., Zhang Y., Liu X. (2022). A Study of the Reliability and Accuracy of the Real-Time Detection of Forage Maize Quality Using a Home-Built Near-Infrared Spectrometer. Foods.

[14] Wang Z., Huang W., Li J., Liu S., Fan S. (2023). Assessment of Protein Content and Insect Infestation of Maize Seeds Based on On-Line near-Infrared Spectroscopy and Machine Learning. Comput. Electron. Agric..

[15] Liu J., Jin S., Bao C., Sun Y., Li W. (2021). Rapid Determination of Lignocellulose in Corn Stover Based on Near-Infrared Reflectance Spectroscopy and Chemometrics Methods. Bioresour. Technol..

[16] Yin C., Deng X., Yu Z., Liu Z., Zhong H., Chen R., Cai G., Zheng Q., Liu X., Zhong J. (2021). Auto-Classification of Biomass through Characterization of Their Pyrolysis Behaviors Using Thermogravimetric Analysis with Support Vector Machine Algorithm: Case Study for Tobacco. Biotechnol. Biofuels.

[17] Xue J., Yang Z., Han L., Liu Y., Liu Y., Zhou C. (2015). On-Line Measurement of Proximates and Lignocellulose Components of Corn Stover Using NIRS. Appl. Energy.

[18] Zhang K., Xu Y., Johnson L., Yuan W., Pei Z., Wang D. (2017). Development of Near-Infrared Spectroscopy Models for Quantitative Determination of Cellulose and Hemicellulose Contents of Big Bluestem. Renew. Energy.

[19] Lestander T.A., Rudolfsson M., Pommer L., Nordin A. (2014). NIR Provides Excellent Predictions of Properties of Biocoal from Torrefaction and Pyrolysis of Biomass. Green Chem..

[20] Triolo J.M., Ward A.J., Pedersen L., Løkke M.M., Qu H., Sommer S.G. (2014). Near Infrared Reflectance Spectroscopy (NIRS) for Rapid Determination of Biochemical Methane Potential of Plant Biomass. Appl. Energy.

[21] Li J., Liu W., Qiu X., Zhao X., Chen Z., Yan M., Fang Z., Li Z., Tu Z., Huang J. (2022). Lignin: A Sustainable Photothermal Block for Smart Elastomers†. Green Chem..

[22] Falcioni R., Gonçalves J.V., Oliveira K.M., Oliveira C.A., Demattê J.A.M., Antunes W.C., Nanni M.R. (2023). Enhancing Pigment Phenotyping and Classification in Lettuce through the Integration of Reflectance Spectroscopy and AI Algorithms. Plants.

[23] Pedro S.I., Antunes C.A.L., Horta C., Pitacas I., Gonçalves J., Gominho J., Gallardo E., Anjos O. (2023). Characterization of Mineral Composition and Nutritional Value of Acacia Green Pods. Plants.

[24] Falcioni R., Antunes W.C., Demattê J.A.M., Nanni M.R. (2023). Reflectance Spectroscopy for the Classification and Prediction of Pigments in Agronomic Crops. Plants.

[25] Kang D.I., Jeong H.K., Park Y.G., Jeong B.R. (2019). Flowering and Morphogenesis of Kalanchoe in Response to Quality and Intensity of Night Interruption Light. Plants.

[26] Gao J., Hao L., Jiang R., Liu Z., Tian L., Zhao J., Ming W., Ren L. (2022). Surprisingly Fast Assembly of the MOF Film for Synergetic Antibacterial phototherapeutics††Electronic Supplementary Information (ESI). Green Chem..

[27] Knez D., Hanninen A.M., Prince R.C., Potma E.O., Fishman D.A. (2020). Infrared Chemical Imaging through Non-Degenerate Two-Photon Absorption in Silicon-Based Cameras. Light Sci. Appl..

[28] Pirutin S.K., Jia S., Yusipovich A.I., Shank M.A., Parshina E.Y., Rubin A.B. (2023). Vibrational Spectroscopy as a Tool for Bioanalytical and Biomonitoring Studies. Int. J. Mol. Sci..

[29] Rasooli N., Farpoor M.H., Mahmoodabadi M., Esfandiarpour-Boroujeni I. (2023). Vis-NIR Spectroscopy as an Eco-Friendly Method for Monitoring Pedoenvironmental Variations and Pedological Assessments in Lut Watershed, Central Iran. Soil Tillage Res..

[30] A R., Mu X., He J. (2022). Enhance Tensor RPCA-Based Mahalanobis Distance Method for Hyperspectral Anomaly Detection. IEEE Geosci. Remote Sens. Lett..

[31] Shaaban-Ali M., Momeni M., Denault A. (2021). Clinical and Technical Limitations of Cerebral and Somatic Near-Infrared Spectroscopy as an Oxygenation Monitor. J. Cardiothorac. Vasc. Anesth..

[32] Choi J., Shin J.G., Kwon H.-S., Tak Y.-O., Park H.J., Ahn J.-C., Eom J.B., Seo Y., Park J.W., Choi Y. (2022). Development of Intraoperative Near-Infrared Fluorescence Imaging System Using a Dual-CMOS Single Camera. Sensors.

[33] de Santana F.B., de Giuseppe L.O., de Souza A.M., Poppi R.J. (2019). Removing the Moisture Effect in Soil Organic Matter Determination Using NIR Spectroscopy and PLSR with External Parameter Orthogonalization. Microchem. J..

[34] Park J., Choi D.H., Jeon Y.-B., Nam Y., Hong M., Park D.-S. (2018). Network Anomaly Detection Based on Probabilistic Analysis. Soft Comput..

[35] Du Z., Tian W., Tilley M., Wang D., Zhang G., Li Y. (2022). Quantitative Assessment of Wheat Quality Using Near-Infrared Spectroscopy: A Comprehensive Review. Compr. Rev. Food Sci. Food Saf..

[36] Vivó-Truyols G., Schoenmakers P.J. (2006). Automatic Selection of Optimal Savitzky−Golay Smoothing. Anal. Chem..

[37] Roger J.-M., Boulet J.-C., Zeaiter M., Rutledge D.N., Brown S., Tauler R., Walczak B. (2020). 3.01—Pre-Processing Methods☆. Comprehensive Chemometrics.

[38] Fearn T., Riccioli C., Garrido-Varo A., Guerrero-Ginel J.E. (2009). On the Geometry of SNV and MSC. Chemom. Intell. Lab. Syst..

[39] Visnupriyan R., Flanagan B.M., Harper K.J., Cozzolino D. (2024). Near Infrared Spectroscopy Combined with Chemometrics as Tool to Monitor Starch Hydrolysis. Carbohydr. Polym..

[40] Peng C., Che Z., Liao T.W., Zhang Z. (2023). Prediction Using Multi-Objective Slime Mould Algorithm Optimized Support Vector Regression Model. Appl. Soft Comput..

[41] Pearson K. (1909). Determination of the Coefficient of Correlation. Science.

[42] Zhao N., Wu Z., Cheng Y., Shi X., Qiao Y. (2016). MDL and RMSEP Assessment of Spectral Pretreatments by Adding Different Noises in Calibration/Validation Datasets. Spectrochim. Acta Part A Mol. Biomol. Spectrosc..

[43] Ai N., Jiang Y., Omar S., Wang J., Xia L., Ren J. (2022). Rapid Measurement of Cellulose, Hemicellulose, and Lignin Content in Sargassum Horneri by Near-Infrared Spectroscopy and Characteristic Variables Selection Methods. Molecules.

[44] Zhou C., Han G., Gao S., Xing M., Song Y., Jiang W. (2018). Rapid determination of cellulose content in pulp using near infrared modeling technique. Bioresources.

[45] Guo T., Dai L., Yan B., Lan G., Li F., Li F., Pan F., Wang F. (2021). Measurements of Chemical Compositions in Corn Stover and Wheat Straw by Near-Infrared Reflectance Spectroscopy. Animals.

